# An Assessment of the "TB Mukt Gram Panchayat" Initiative in Northeastern Madhya Pradesh, India

**DOI:** 10.7759/cureus.112115

**Published:** 2026-07-05

**Authors:** Pushpendra Sahu, Yogesh Shukla, Pooja Gupta, Ambrish Mishra, Shailendra Singh

**Affiliations:** 1 Community Medicine, Government Medical College Satna, Satna, IND; 2 District Tuberculosis Centre, District Hospital Satna, Satna, IND

**Keywords:** tb elimination policy, tb-free village council, tb-free villages, tuberculosis control, tuberculosis elimination

## Abstract

Introduction: After more than 50 years of control activities directed towards tuberculosis (TB), it is still a severe health crisis in India. India has committed to achieving the end TB target by 2025. To monitor the progress of TB elimination in India, the Government of India implemented a decentralised plan to achieve the goal of TB elimination at the country level. Every village should reach TB-free status. For this, the *TB Mukt* *Gram Panchayat* (TB-free village) initiative is presently being promoted. For these substantial efforts in ending TB in India, these efforts need to be measured and validated to declare *gram panchayats* (villages)* TB mukt* (TB-free). This research paper explains the implementation process for the validation of *TB mukt panchayats*, with challenges faced in the process.

Methodology: We evaluated the process of declaring *panchayats* TB-free in the Satna and Maihar districts over the past three years. In 2024, seven and one *gram panchayats* were nominated from the Satna and Maihar districts, respectively; in 2025, 14 and 11 *gram panchayats* were nominated, respectively; and in 2026, 162 and 117 *gram panchayats* were nominated from the Satna and Maihar districts, respectively. The assessment process was conducted by a district-level team that was formed to verify the six indicators and the TB-free claim in the 1^st^ quarter of every year. The team gathered, triangulated, and collated data from various sources, and the findings were evaluated. Certificates for "*TB Mukt Panchayat*" with one-year validity and a small statue of Mahatma Gandhi are to be awarded to qualified *gram panchayats*.

Results: While verifying the submitted claims, the claim success rate in the Satna district was 83.33% in 2024, 85.72% in 2025, and 77.78% in 2026. In Maihar district, no *gram panchayats* were verified against the claim of a TB-free *gram panchayat* in 2024. In 2025 and 2026, claim success rates of 45.45% and 80.34%, respectively, were observed. From all six indicators, the TB notification rate was the most common indicator that hindered getting TB-free *panchayat* ​​​​​​status.

Conclusion: As this study indicates that progress has occurred over the years across all six indicators used to declare *gram panchayats* TB-free, public health response and participation have increased gradually over the past three years, and *gram panchayats* have become more eager to claim TB-free status, which will help India eliminate TB. This study will help the programme evaluators to assess the TB-free *gram panchayat* status in the future.

## Introduction

After more than 50 years of control activities directed towards tuberculosis (TB), TB is still a severe health crisis in India. The incidence of TB in India is 199 per lakh population, and the mortality rate was 23 per lakh population in 2022 [1].

India has committed to achieving the end TB target by 2025. To achieve these targets, the National Tuberculosis Elimination Programme implemented the National Strategic Plan (NSP) 2017-2025 for TB elimination in India [2]. Community engagement and the engagement of local self-government bodies, such as Panchayati Raj Institutions (PRIs), are intended to leverage the trust of the PRIs and the local community, which is a key strategy [3]. The Ministry of Health launched the *TB Mukt Bharat Abhiyan* (TB-Free India Initiative), calling for a *Jan Andolan* or people’s movement to generate demand for TB services in the community [4].

To monitor the progress of elimination of TB in India, the Government of India (GOI) implemented multiple plans of action, such as the launch of sub-national certificates in 2021 to incentivise and reward the best-performing districts/states and motivate them to achieve the targets of TB elimination [5]. To further decentralise efforts to achieve the goal of TB elimination at the country level, every village and *panchayat* (village council) should achieve TB-free status. For this, the *TB Mukt Gram Panchayat *(TB-free village) is presently being promoted as a major strategy for scaling up initiatives aimed towards TB elimination from India and to empower the community to realise the extent and magnitude of the problems associated with TB and take necessary actions towards solving the problems for reducing the burden of TB, as well as achieving a relapse-free cure for all TB patients. The Hon’ble Prime Minister of India launched the *TB Mukt Gram Panchayat* initiative at the One World TB Summit in 2023 in Varanasi to leverage the support of 2.5 lakh *gram panchayats* towards the elimination of TB [6].

For these substantial efforts in ending TB in India, these efforts need to be measured and validated to declare the *panchayats* *TB mukt *(TB-free).

This research paper explains the implementation process for the validation of *TB Mukt Panchayats*, with challenges faced in the process.

Objective

To evaluate the TB-free status of nominated *gram panchayats* of Satna and Maihar districts based on the six indicators suggested by the Central TB Division, India, over the last three years.

## Materials and methods

Study design

The study was designed as a community-based observational study.

Setting

Satna is a northeastern city of Madhya Pradesh, which has five blocks/*janpad panchayats* and 449 *gram panchayats*. Maihar is the latest district in Madhya Pradesh, carved out of Satna district, with three blocks/*janpad panchayats* and 254 *gram panchayats* [7,8]. The assessment process of both districts was conducted by the district tuberculosis unit of Satna, as the administrative control of health was still governed by Satna district. In 2024, eight *gram panchayats* (seven from Satna and one from Maihar district) were nominated from four blocks (three blocks of Satna and one from Maihar district) for TB elimination; in 2025, 16 *gram panchayats* were nominated from three blocks of Satna and 11 *gram panchayats* from two blocks of Maihar district; in 2026, 162 *gram panchayats* were nominated from Satna district, comprising all blocks, and 117 from Maihar district, from three blocks.

Participants

A total of 314 *gram panchayats* (eight blocks) were selected based on nominations submitted by *gram panchayats* (blocks) to declare *panchayats* TB-free for the year based on indicators mentioned in Table 1 [9].

**Table 1:** List of indicators and targets for TB Mukt Panchayat claim to be verified (for year 2025). TB: tuberculosis; DS-TB: drug-susceptible tuberculosis; CBNAAT: cartridge-based nucleic acid amplification test. Source: Central TB Division [9].

S. No.	Indicator	Description	Target
1.	Number of presumptive TB examination/1000 populations	Numerator: Number of presumptive TB persons examined in the year x 1000	30 per 1000 population (for the year) in the *panchayat*.
		Denominator: Total population of the *panchayat* in the year	
2.	TB notification rate/1000 population	Numerator: Numerator of TB cases notified (all types) in the year x 1000	1 per 1000 population (for the year) in the *panchayat*.
		Total population of the *panchayat* in the year	
3.	Treatment success rate	Numerator: Number of DS-TB patients notified during the same period of the previous year with treatment outcome given as success, i.e., either cured or treatment completed x 100	85%
		Denominator: Net DS-TB patients notified during the same period of the previous year.	
4.	Drug susceptibility test rate	Numerator: Number of TB patients with an available result for at least rifampicin in the year (offered TrueNat and CBNAAT)	At least 60%
		Denominator: Number of TB patients notified in the year in the *panchayat*.	
5.	Nikshay Poshan Yojana	Numerator: Number of TB patients who paid at least one instalment in the year.	50%
		Denominator: Number of eligible TB patients in the year in the *panchayat*.	
6.	Nutritional support to TB patients under the *Pradhan Mantri TB Mukt Bharat Abhiyan* (PMTBMBA)	Numerator: Number of TB patients who received nutrition support under PMTBMBA in the year.	100%
		Denominator: Number of TB patients consented to receive the nutritional support under PMTBMBA in the year in the *panchayat*.	

Variables

Seven outcome variables were as follows: number of presumptive TB examination/1000 populations; TB notification rate/1000 population; treatment success rate; drug susceptibility test rate; *Nikshay Poshan Yojana*; and nutritional support to TB patients under the *Pradhan Mantri TB Mukt Bharat Abhiyan* (Prime Minister's Tuberculosis-Free India Campaign; described in Table 1); and overall claim success percentage comprising all the six indicators.

Data sources

Quantitative data were gathered from nucleic acid amplification test (cartridge-based nucleic acid amplification test (CBNAAT) and TrueNat) lab registers, notification register, registers from public-private support agencies, and online portals such as *Nikshay* (using different modules, i.e., notification, lab, Nikshay Mitra, direct benefit transfer). Qualitative data were gathered from interviews of chemists from nearby health facilities, telephonic conversations with village heads (*sarpanch*), community health officers (CHOs), Accredited Social Health Activists (ASHA), and notified TB patients, and used for confirmation and cross-check of quantitative data. Triangulation and collation of the data were done to assess the verification of all six indicators.

The key contributors for providing the necessary data are the senior treatment supervisor (STS), senior tuberculosis lab supervisor (STLS), lab technician, medical officer - TB control (MO-TC), district program coordinator (DPC), and data entry operator.

Bias

As one of the coauthors is a program implementer, confirmation bias may occur. However, this potential bias was minimised through the study methodology, as the other authors conducted the data collection and verification processes, while the program implementer was not directly involved in the verification process.

Study size

All 314 *gram panchayats* from eight blocks of Satna and Maihar districts were included, which were self-nominated to declare the *panchayats* TB-free.

Quantitative variables

Quantitative variables were collated and gathered according to study objectives and summarised as frequency, percentage, and confidence interval.

Study procedure

The assessment process of both districts was conducted by the district tuberculosis unit of Satna. To declare any panchayat *TB mukt*, capacity-building meetings are held at the start of the evaluation process, with the participation of the district and all blocks, with the presence of the district tuberculosis officer (DTO) and district Panchayati Raj officer (DPRO). After that, *panchayats* prepare a plan to achieve *TB Mukt Panchayat* status. They include *TB Mukt* activities in *panchayat* development plans (PDPs) to raise awareness about the various aspects of TB, monitor quarterly reports using the checklist during *Jan Arogya Samiti* and Village Health and Sanitation Committee meetings, and provide nutritional support as part of the *Pradhan Mantri TB Mukt Bharat Abhiyan* with the support of the district TB centre.

A block tuberculosis unit assesses the *panchayat* claim and submits it to the district tuberculosis unit with all relevant documents for claim verification. The DTO submits the claim to the state and further to the central tuberculosis unit. The process of verification starts with the sensitisation meeting, where the DTO gives insights into the verification process and introduces various staff of the tuberculosis unit at the district and block levels, and helps maintain coordination for the further process [10].

Every year, a district-level team was formed to verify the submitted indicators listed in Table 1 and the TB-free claims during the first quarter of the year. The team comprised the Chief Medical & Health Officer (CM&HO) as the chairperson, the DTO as the convener or a representative of the State Tuberculosis Officer, a representative from the district *panchayat*, a member of the Indian Association of Preventive and Social Medicine/Department of Community Medicine, a medical college faculty member, a member of the Indian Medical Association, and a postgraduate medical officer (PGMO)/medical officer (MO).

The verified list of *gram panchayats* that qualify for *TB Mukt Panchayat* status is to be submitted by the district TB team to the district magistrate/district collector/deputy commissioner and to the state tuberculosis office through the district tuberculosis centre. State- and district-wise reports of *TB Mukt Gram Panchayats* are to be generated by states and shared with the Ministry of Panchayati Raj (MoPR) and Central TB Division (CTD), Ministry of Health and Family Welfare (MoHFW), in March every year.

Certificates for *TB Mukt Panchayat* with one year's validity and a small statue of Mahatma Gandhi to be awarded are issued to qualified *gram panchayats* on World TB Day, 24th March [10].

Statistical methods

Data were cleaned in Microsoft Excel (Microsoft Corporation, Redmond, WA), and descriptive statistics of the data are presented in frequency tables and percentages with confidence intervals according to blocks and *gram panchayats*.

## Results

In Satna district, for the year 2023, seven *gram panchayats* from three blocks (Sohawal, Unchehara, and Nagod) submitted claims for *TB Mukt Gram Panchayat* status. For the year 2024, 14 *gram panchayats* from the same three blocks submitted claims, and for the year 2025, 162 *gram panchayats* from five blocks (Sohawal, Unchehara, Nagod, Majhgawan, and Rampur Baghelan) submitted claims for TB-free status.

While verifying submitted claims in 2024, six *gram panchayats* were verified against the TB-free *gram panchayat* claim, with an 83.33% claim success rate. In 2025, 12 *gram panchayats* were verified against the submitted claim, with an 85.72% success rate. In the year 2026, 126 *gram panchayats* were successfully verified for TB-free status, with a 77.78% claim success rate, as shown in Table 2.

**Table 2:** Verification status of gram panchayats claiming the status of TB-free for the last three years. TB: tuberculosis.

Claim verification year	Blocks	*Gram panchayat* claimed TB-free status	*Gram panchayat* verified as TB-free	Claim success (%) ± (95% CI)	Key indicator unachieved (not mutually exclusive)					
					Number of presumptive TB examinations	TB notification rate	Treatment success rate	Drug susceptibility test rate	Nikshay Poshan Yojana	Nutritional support to TB patients under the *Pradhan Mantri TB Mukt Bharat Abhiyan*
District Satna										
2024	Sohawal	04	04	100 ±(0.0)	NA	NA	NA	NA	NA	NA
	Unchehara	02	01	50 ± (69)	01	01	00	00	00	01
	Nagod	01	01	100 ± (0)	NA	NA	NA	NA	NA	NA
2025	Sohawal	06	05	83 ± (30)	01	00	01	00	00	00
	Unchehara	01	01	100 ± (0)	NA	NA	NA	NA	NA	NA
	Nagod	07	06	86 ± (26)	00	01	00	00	00	00
2026	Sohawal	49	42	86 ± (10)	04	04	00	00	02	02
	Unchehara	16	14	88 ± (16)	00	02	00	00	00	00
	Nagod	31	22	71 ± (16)	01	07	01	00	00	00
	Majhgawan	17	13	76 ± (20)	00	04	00	01	00	00
	Rampur Baghelan	49	35	71 ± (13)	02	14	00	02	04	00
District Maihar										
2024	Ramnagar	01	00	0 ± (0)	01	01	NA	NA	01	NA
2025	Ramnagar	01	01	100 ± (0)	NA	NA	NA	NA	NA	NA
	Amdara	10	04	40 ± (30)	00	06	NA	NA	NA	NA
2026	Ramnagar	24	19	79 ± (16)	00	05	00	00	00	00
	Amdara	49	40	82 ± (11)	02	09	00	00	00	00
	Amarpatan	44	35	80 ± (12)	05	06	00	00	00	00

In Maihar district, for the year 2023, only one *gram panchayat* from one block (Ramnagar) submitted a claim for *TB Mukt Gram Panchayat* status. For the year 2024, 11 *gram panchayats* from two blocks (Ramnagar and Amdara) submitted a claim, and for the year 2025, 117 *gram panchayat*s from three blocks (Ramnagar, Amdara and Amarpatan) submitted claims for TB-free status.

While verifying submitted claims in 2024 for Maihar district, no *gram panchayats* were verified against the claim of a TB-free *gram panchayat*. In 2025, five *gram panchayats* were verified against the claim, with a 45.45% success rate, and in the year 2026, 94 *gram panchayats* were successfully verified for TB-free status, with an 80.34%% claim success rate, as shown in Table 2.

As the six indicators defined in Table 1 were verified against the submitted claims in Satna district for the year 2024, the number of presumptive TB examinations, the TB notification rate, and the provision of nutritional support to TB patients under the *Pradhan Mantri TB Mukt Bharat Abhiyan* did not meet the criteria for TB-free status of the *gram panchayat*. In the year 2025, the number of presumptive TB examinations, the TB notification rate, and the treatment success rate were not verified. In 2026, among the 36 disqualified *gram panchayats*, 31 (86.11%) were not verified for the TB notification rate as an elimination indicator, seven (19.44%) were not verified for the number of presumptive TB examinations, six (16.67%) were not verified for the *Nikshay Poshan Yojana*, two (5.55%) were not verified for nutritional support to TB patients under the *Pradhan Mantri TB Mukt Bharat Abhiyan*, and one (2.78%) failed verification for the treatment success rate indicator supporting TB-free status, as indicated in Table 2.

For Maihar district in the year 2024, the number of presumptive TB examinations, the TB notification rate, and the *Nikshay Poshan Yojana* were not verified and do not support the criteria for TB-free status of the *gram panchayat*. In the year 2025, the TB notification rate was the only indicator that was not verified and did not support the criteria for TB-free status of *gram panchayat*, and in 2026, 20 *gram panchayats* from 23 disqualified *gram panchayats* (86.95%) were not verified for the TB notification rate as an elimination indicator, seven *gram panchayats* (30.43%) failed verification for the number of presumptive TB examinations as an indicator supporting TB-free status of the *gram panchayat*, and none of the other indicators obstructed the pathway to TB-free claim status for the *gram panchayats*, as shown in Table 2.

In 2026, six *gram panchayats* were not verified for both the number of presumptive TB examinations and the TB notification rate as elimination indicators; four *gram panchayats* were not verified for the TB notification rate and the *Nikshay Poshan Yojana*; three *gram panchayats* were not verified for the TB notification rate and the drug susceptibility test rate; one *gram panchayat* was not verified for the TB notification rate, the *Nikshay Poshan Yojana*, and nutritional support to TB patients under the *Pradhan Mantri TB Mukt Bharat Abhiyan*; and one *gram panchayat* was not verified for the TB notification rate, the *Nikshay Poshan Yojana*, nutritional support to TB patients under the *Pradhan Mantri TB Mukt Bharat Abhiyan*, and the number of presumptive TB examinations as combined indicators in both districts, and therefore did not support the claim for TB-free status of the *gram panchayats*.

**Figure 1:**
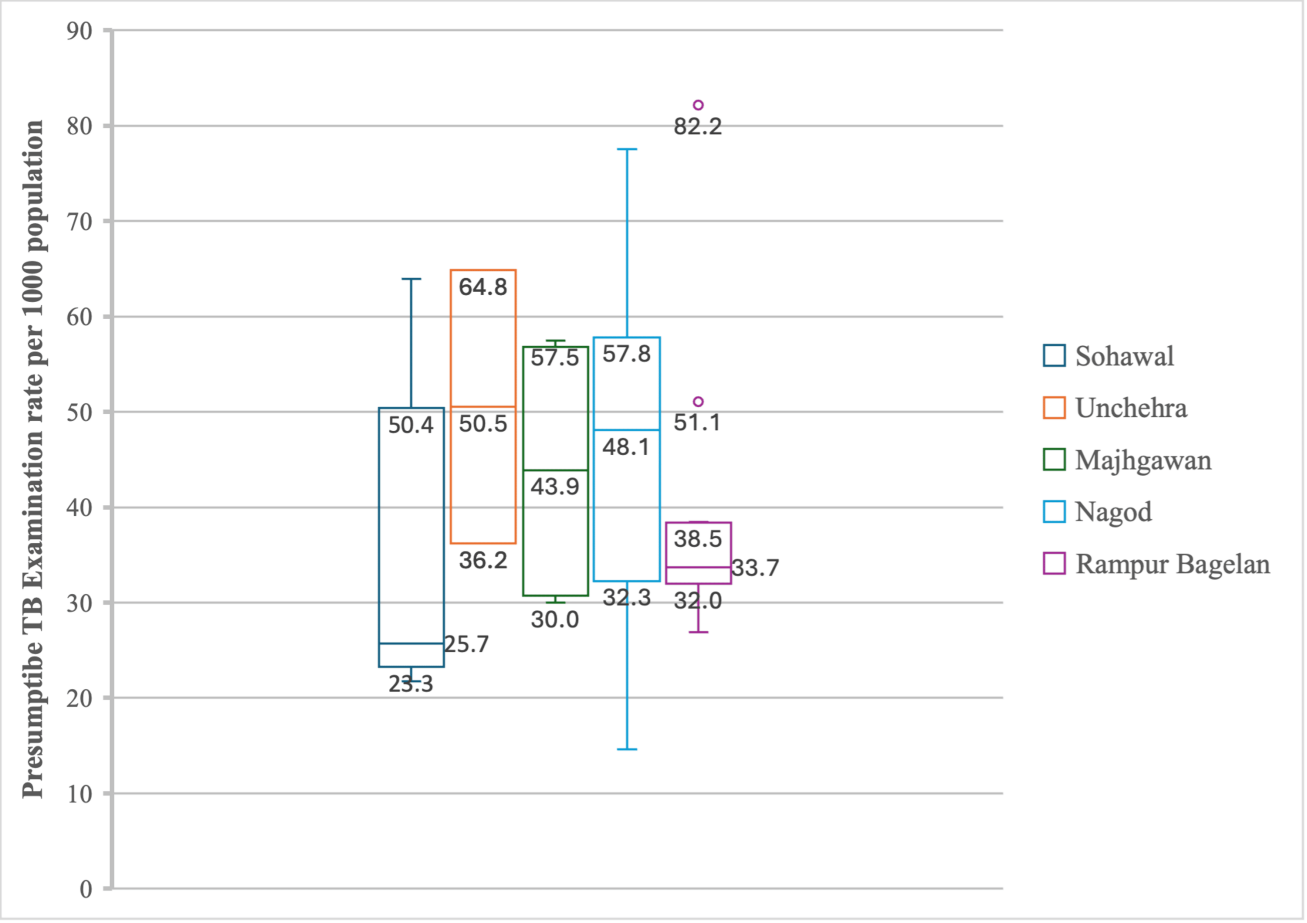
Comparison of presumptive tuberculosis (TB) examination rate among panchayats that did not pass the TB-free criteria, comprising blocks of Satna district.

Figure 1 represents the comparison of the presumptive TB examination rate per 1,000 population among *panchayats* that did not comply with the TB-free criteria across the blocks of Satna district. The figure shows that the *panchayats* of Sohawal block had a median rate of 25.7, which was below the TB-free criterion of 30 per 1,000 population, compared with the other blocks in the district. The variation in the testing rate was highest in the *panchayats* of Nagod and Rampur Baghelan blocks, with median (range) values of 48.1 (14.6-77.6) and 33.7 (26.88-82.2), respectively, compared with the other blocks in Satna district.

In Maihar district, the Amarpatan block shows the highest variability with a median of 9.3 and a range of 5.1 to 142.3.

**Figure 2:**
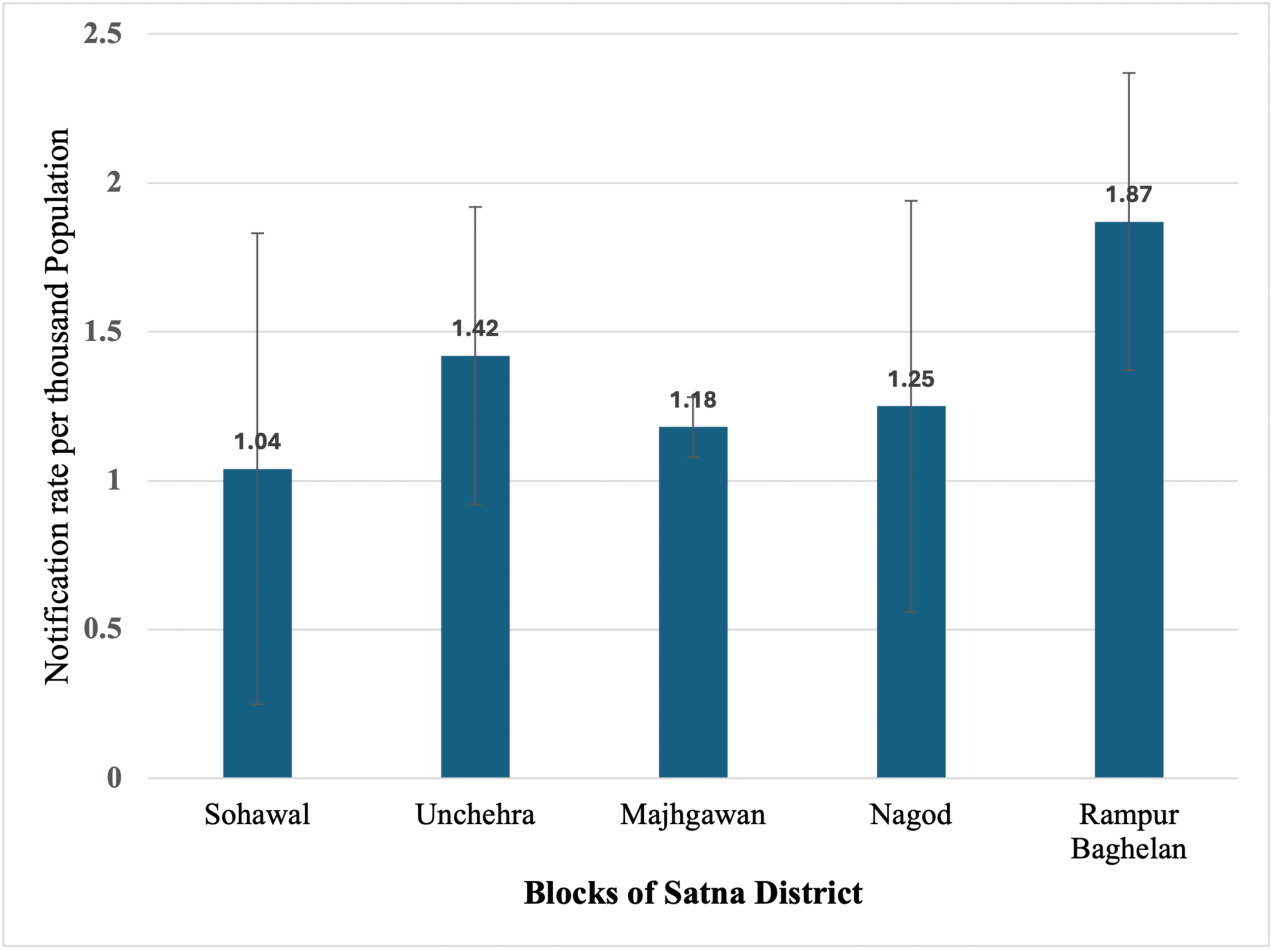
Comparison of notification rates of panchayats that did not pass the TB-free criteria, comprising the blocks of Satna district. TB: tuberculosis.

**Figure 3:**
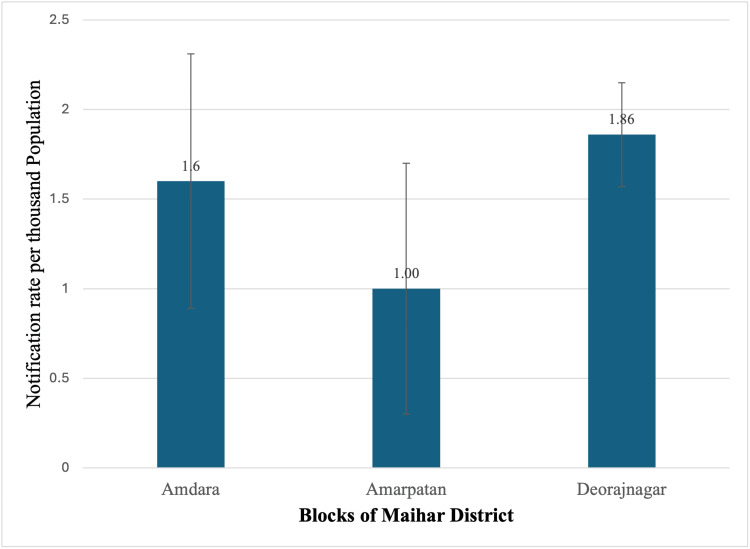
Comparison of the notification rate of panchayats that did not pass the TB-free criteria, comprising the blocks of Maihar district. TB: tuberculosis.

Figures 2, 3 show the comparison of the presumptive TB notification rate per 1,000 population among *panchayats* that did not comply with the TB-free criteria across the blocks of Satna and Maihar districts. As shown, all blocks in Satna and Maihar districts had a mean that is above the criterion of the TB notification rate for TB-free status. The highest mean TB notification rate in Satna district was observed in Rampur Baghelan block (1.87 ± 0.5), followed by Unchehara block (1.42 ± 0.5), while in Maihar district, Deorajnagar (1.86 ± 0.29) and Amdara (1.60 ± 0.71) blocks showed high mean notification rates. These findings indicate that the TB notification rate is a key criterion that needs to be prioritised.

## Discussion

India has set the ambitious target to eliminate TB from India by 2025, five years ahead of the global target [1]. To achieve this, community engagement is identified as a key strategy. Community engagement has been broadly defined as "involving communities in decision-making and in the planning, design, governance, and delivery of services," and it is implemented through various methods and at various levels [11]. In India, to make the *gram panchayat* TB-free, the *gram pradhan* (village council head) is identified as a key player to enhance community engagement [9]. Different methods to increase community engagement for TB awareness are used and found beneficial, as reported by Quraishi et al. in 2022 [12], and can be incorporated at the *panchayat* level.

Also, an active case-finding campaign that mobilises almost the entire general health system to conduct house-to-house screening for TB symptoms among mapped vulnerable target populations multiple times a year is another strategy that directly contributes to achieving TB-free *panchayat* status. ﻿Active case finding (ACF) activities were initiated in India in 2009 by nongovernmental organisations (NGOs) [13], which can decrease the TB incidence in the future, according to a modelling study conducted by Ayabina et al. [14].

This study comprises three years of activities in two districts of Madhya Pradesh and gives an insight into the *TB Mukt Gram Panchayat* campaign. As this study clearly indicates, like other community engagement activities, public health response and participation are increasing gradually. In 2024, only eight *gram panchayats* participated, but in 2026, 279 *gram panchayats* claimed and participated to make *panchayats* TB-free. This clearly indicates that coordination and collaboration are increased between the district-level team and PRIs.

Claim success percentage is also increased from 62.5% in the year 2024, which is similar to the success percentage mentioned in the study conducted by Bhardwaj et al. in Rajasthan, to 89.8%, which was conducted in 51 *gram panchayats* for evaluation in the year 2023 for the same six indicators evaluated in this study [15]. A clear target and motivation help the field-level team of the National TB Elimination Program (NTEP) to strengthen their performance and align their objective with the indicators of the TB-free status claim.

The TB notification rate has come out as the key indicator for not achieving the TB-free status by the *gram panchayats*, because there is difficulty in the alignment between digital (Excel sheet of Nikshay Portal) and physical records (notification registers and public-private partnership (PPP)-maintained registers), as this is the critical criterion that affects TB transmission.

Universal drug sensitivity testing is also improved because of decentralisation and innovation in drug susceptibility testing and increased availability of CBNAAT and true nucleic acid amplification testing machines at the peripheral level, which support the two indicators (number of presumptive TB examinations and drug susceptibility test rate) and help early identification of drug resistance and TB treatment guidance [16] that directly affect the criteria of treatment success rate. Some blocks (Nagod, Rampur Baghelan, and Amarpatan) in both districts show high variability between presumptive tests conducted; this variability needs to be normalised so that more resources can be equitably distributed.

This TB-free *gram panchayat* activity is increasing its effect since its launch and is helping the community leaders to increase awareness in the community as a key task of community involvement and a good example of intersectoral collaboration, and strengthening the pillar of the National Tuberculosis Elimination Programme [2]. Also, achieving the TB-free status certificate and receiving the statue of Shri Mahatma Gandhi is a key motivator to achieve this task.

While this activity helps the overall goal of eliminating TB from India in future, some areas still need to be strengthened: (a) as active case-finding of TB cases is very important to achieve the TB-free status, the quality needs to be maintained for the collection of samples and the sampling population, as mentioned by the senior TB laboratory supervisor while interviewing, that the quality of samples is not satisfactory. (b) Streamlined and timely payment instalment of the *Nikshay Poshan Yojana* needs to be strengthened, as mentioned by the beneficiary, that the DBT transfer is not regular, and confirmed by the data entry operator that hindrances occur at various levels, for example, at the bank verification level, as regular mergers of banks are ongoing, which leads to changes in the Indian Financial System Code (IFSC). (c) More staff/cadre need to be engaged in the activity to decrease the already burdened staff of the programme, so that more *panchayats* can be nominated to achieve TB-free *gram panchayat* status. (d) There are multiple provisions to give support to TB patients under the *Pradhan Mantri TB Mukt Bharat Abhiyan*, but narrowly focused on nutrition support (food basket), which is not achieving the overall goal, as it is observed that only food basket is provided to the patient under the *Pradhan Mantri TB Mukt Bharat Abhiyan* while interviewing the patient and *gram panchayat sarpanch*, besides skill development and other benefits.

More similar studies in India are needed to generalise the findings, as very few studies have been conducted to date.

## Conclusions

This study provides insight into the current activities followed under the *TB Mukt Gram Panchayat* initiative through the evaluation process conducted in some districts of Madhya Pradesh, indicating that progress has occurred across all six indicators, i.e., the number of presumptive TB examination/1000 populations, TB notification rate/1000 population, treatment success rate, drug susceptibility test rate, *Nikshay Poshan Yojana*, and nutritional support to TB patients under the* Pradhan Mantri TB Mukt Bharat Abhiyan* to declare *panchayats* TB free. Also, it indicates that community engagement is increasing, as *gram panchayats* are becoming more eager to claim TB-free status with each passing year, which will help India eliminate TB. This study will help the programme evaluators to assess the TB-free *gram panchayat* status in the future.
